# Managing lorlatinib-induced weight gain through a structured exercise intervention in an ALK+ NSCLC patient: a case report

**DOI:** 10.3389/fonc.2025.1672319

**Published:** 2025-11-20

**Authors:** Christian Ciurnelli, Ilaria Mariangela Scaglione, Serena Eccher, Anita Borsati, Linda Toniolo, Gloria Adamoli, Daniela Tregnago, Lucia Longo, Marco Sposito, Jessica Insolda, Michele Milella, Federico Schena, Morten Quist, Lorenzo Belluomini, Sara Pilotto, Alice Avancini

**Affiliations:** 1 Department of Neurosciences, Biomedicine and Movement Sciences, University of Verona, Verona, Italy; 2 Section of Oncology, Department of Engineering for Innovation Medicine, University of Verona School of Medicine and Verona University Hospital Trust, Verona, Italy; 3 Biomedical, Clinical and Experimental Sciences, Department of Medicine, University of Verona, Verona, Italy; 4 Rigshospitalet, University Hospital of Copenhagen, Copenhagen, Denmark

**Keywords:** lorlatinib, weight gain, lung cancer, physical exercise, weight management, body composition

## Abstract

**Background:**

In non-small cell lung cancer (NSCLC), ALK tyrosine kinase inhibitors (TKIs) have been associated with significant weight gain, predominantly in fat tissues. To date, only one case report has evaluated the impact of pharmacologic intervention on managing this drug-related side effect, with concerns about potential interactions with oncological treatments. No information is available regarding non-pharmacological intervention.

**Case description:**

A 43-year-old man affected by metastatic ALK-rearranged NSCLC developed Grade 1 weight gain 1.5 months after starting Lorlatinib. The patient participated in a structured exercise program to manage this side effect. The program lasted 6 months and consisted of aerobic activities, including continuous and interval training, as well as resistance exercises using body weight/isotonic machines, performed at moderate to somewhat vigorous intensity, twice a week. Assessments were performed at three and six months, and included physical fitness and patient-reported outcomes parameters. Although the weight remained stable, body composition analysis, via bioimpedance and CT scan, revealed a decrease in fat tissue and an increase in muscle mass. Moreover, the patient reported improvements in physical fitness, especially in cardiorespiratory fitness, muscle strength, endurance, and flexibility. Among the patient-reported outcomes, different domains of quality of life improved.

**Conclusion:**

This case may represent the backbone for further interventional studies aimed at determining the real efficacy of exercise intervention in preventing or controlling weight gain in this population.

## 1 Introduction

For a long time, advanced non-small cell lung cancer (NSCLC) has remained a fearsome disease, in which the unique treatment option was represented by platinum-based chemotherapy (1). Over the past few decades, alongside “*the immunotherapy revolution*”, the discovery of gene alterations has led to the identification of a distinct subgroup of NSCLC, known as “*oncogene-addicted*”. In this specific subgroup, the introduction of tyrosine kinase inhibitors (TKIs) has substantially changed the treatment paradigm for NSCLC harboring specific genetic alterations, offering patients a significant increase in survival and, at the same time, a better quality of life, given the favorable profile in terms of toxicities compared to conventional chemotherapy (2). Consequently, international clinical practice guidelines endorse comprehensive molecular profiling in patients with metastatic NSCLC to detect clinically actionable genomic alterations, thereby driving the selection of targeted agents approved for use in frontline or later-line therapeutic regimens (3). Among these, the rearrangements in the anaplastic lymphoma kinase (ALK) gene account for 3-7% of NSCLC and are more frequently observed in young female, never or light-smoking patients, and with adenocarcinoma histology. Different generations of ALK inhibitors have been tested, showing greater efficacy and overall fewer severe adverse events (2). However, metabolic side effects, such as hypertriglyceridemia and hypercholesterolemia, are frequently observed in patients receiving ALK TKIs, often necessitating lipid-lowering drugs (4). Nevertheless, another potential toxicity, particularly related to the newer generation of ALK TKIs, has emerged: weight gain (5). Our recent meta-analysis has evaluated the weight gain induced by TKIs in NSCLC. Overall, 18 pivotal trials and a total of 3,514 patients were included in the analysis. The results showed that alectinib and crizotinib were associated with a considerable incidence of drug-related weight gain, 15% and 5%, respectively. Still, lorlatinib was reported to have a higher risk of this treatment-related side effect, with an overall incidence of 36%, mainly of mild grade (6). Additionally, recent evidence suggests that the weight gain was primarily due to an increase in adipose tissue (7). This is particularly alarming, since weight gain may profoundly impact patients’ physical and mental well-being, and is additionally a recognized risk factor for several other chronic diseases, e.g., cardiovascular or metabolic disorders. For instance, a prospective observational study, including 123.750 subjects, found that the incidence of metabolic disease (such as diabetes), cardiovascular problems, e.g., hypertension, heart disease, stroke, and also colon cancer, increased with the degree of overweight (8).

To manage this side effect, pharmacologic interventions have been preliminarily proposed. While glucagon-like peptide-1 receptor agonists (GLP-1 RAs) have been demonstrated to be efficacious in weight control in non-cancer populations (2), to date, only one case report assessed the effect of GLP-1 RAs on alectinib-induced weight gain. Despite the effective weight management, it was simultaneously observed that the TKI exposure decreased by one-third, potentially suggesting a negative effect on anti-cancer treatment efficacy (9). Further studies are required to explore the potential interactions between GLP-1Ras and TKIs, but at the same time, other options may be available to manage weight gain in patients with cancer. Physical exercise is essential for determining the caloric balance and thus shaping body composition. In patients with lung cancer, such interventions have been investigated, demonstrating a favorable safety profile and efficacy in improving physical function, as well as benefits in managing various symptoms and side effects, and in modulating muscle mass and adipose tissue (10). However, despite the strong rationale, to our knowledge, no studies have explored exercise as a potential intervention to manage TKIs-induced weight gain. In this context, we report the impact of a 6-month intervention combining tailored exercise in a patient affected by advanced ALK+ NSCLC, as treatment for counteracting lorlatinib-induced weight gain.

## 2 Case description

**Figure 1:**
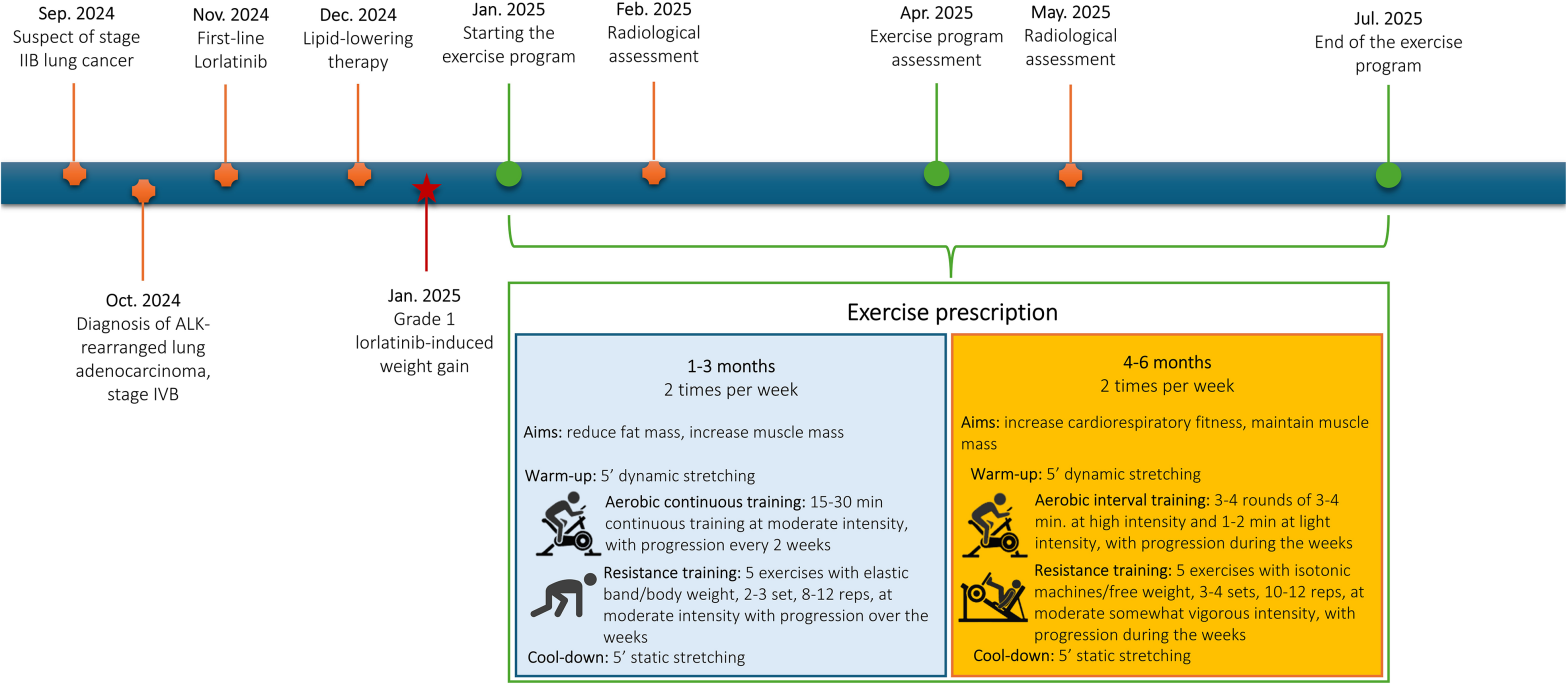
Timeline of disease status and physical exercise intervention.

**Figure 2:**
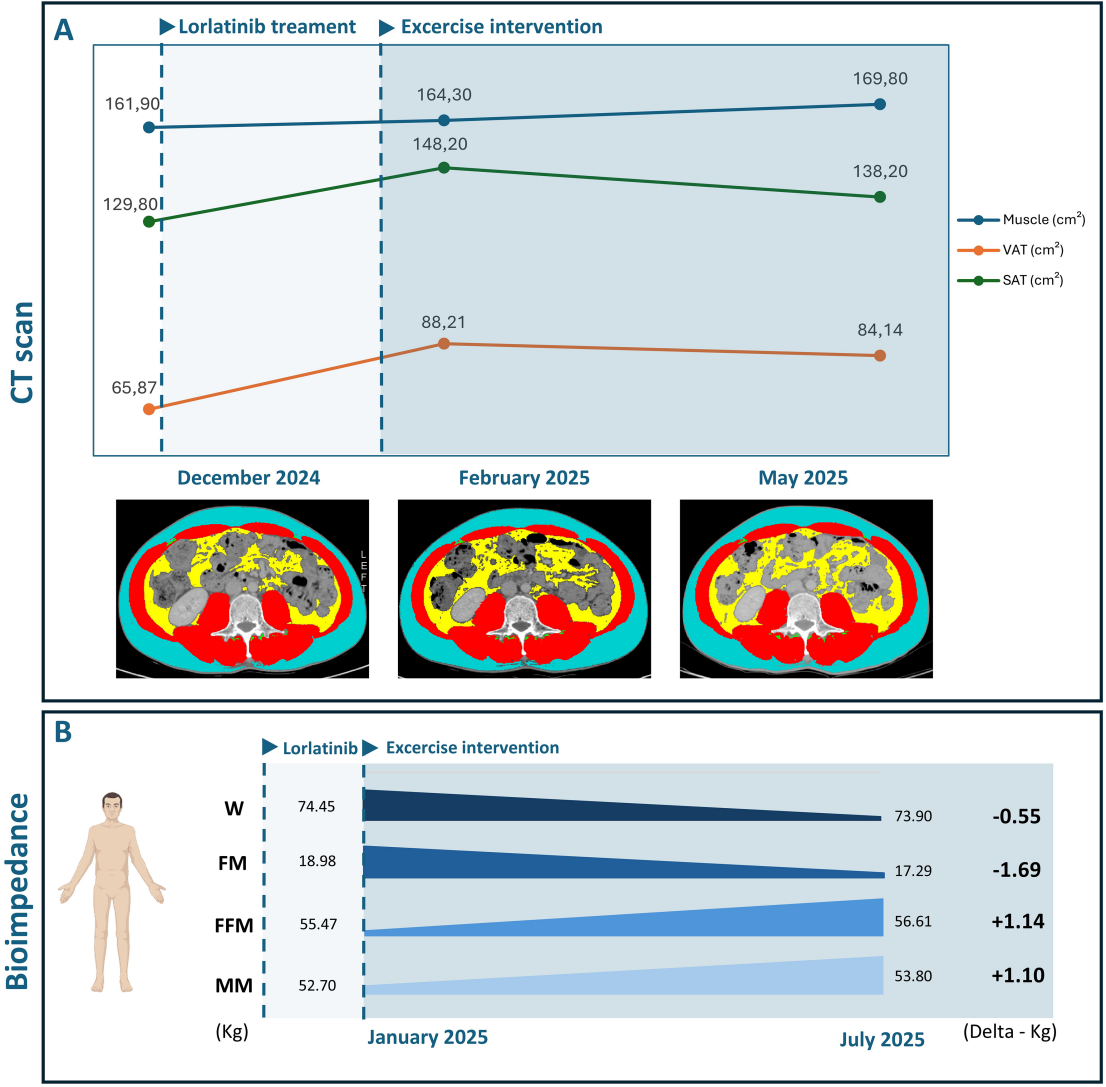
**(A)** Body composition changes since diagnosis and during exercise intervention, assessed via CT scans. **(B)** Body composition changes during exercise intervention, assessed via bioimpedance analysis.

In September 2024, a 43-year-old never-smoker man of Asian ethnicity and without relevant comorbidities, due to the onset of dyspnea and persistent cough, underwent a contrast-enhanced chest CT scan, which revealed a solid 6 × 4 cm nodule in the left lower lobe. Staging was completed with a contrast-enhanced brain CT and 18F-FDG PET scan, which confirmed the presence of the pulmonary lesion without evidence of distant metastases [stage IIB (cT3 N0 M0), according to the 8th edition of the TNM classification]. Histological typing was performed via tracheobronchoscopy, which confirmed the diagnosis of ALK-rearranged lung adenocarcinoma, detected by next-generation sequencing (NGS). In October 2024, during exploratory thoracoscopy, metastatic involvement of the pleura and diaphragm was identified and subsequently confirmed by intraoperative frozen section histological examination. Consequently, lobectomy was deferred. Subsequent CT total body imaging revealed multiple pleural lesions [stage IVB (cT3 Nx M1c] and first-line targeted therapy with lorlatinib 100 mg/day was initiated in November 2024 (**Figure 1**). At the 4-week follow-up, a grade 1 elevation in both cholesterol (214 mg/dL) and triglycerides (246 mg/dL) was observed, prompting the initiation of lipid-lowering therapy (rosuvastatin 5 mg and fenofibrate 145 mg). Approximately 1.5 months after starting lorlatinib, the patient had gained 6 kilograms (kg) of body weight, resulting in a grade 1 weight gain. CT scans at 3 and 6 months showed a reduction in all known disease lesions, with a partial response, according to RECIST 1.1, as the best response. Body composition analysis through CT scan revealed a considerable increase in visceral and subcutaneous adipose tissue after 3 months of lorlatinib treatment compared with the baseline evaluation (**Figure 2A**). The patient continues lorlatinib with good tolerance, but to manage the side effect of weight gain, was referred to the specialized exercise service. In this light, in January 2025, the patient was invited to participate in a 6-month exercise intervention.

This report was conducted following the Declaration of Helsinki and Good Clinical Practice standards and written according to the case report guidelines (CARE guidelines) (11). The authors obtained the patient’s informed consent for the publication of clinical data, guaranteeing the anonymity of personal information.

### 2.1 Intervention

The main goal of the exercise intervention was primarily to manage the lorlatinib-induced weight gain. Additionally, the intervention aimed to alleviate potential symptoms and treatment-related side effects while also enhancing physical function, quality of life, and overall well-being.

### 2.2 Physical exercise

Exercise intervention has been delivered at the Sport Science University facility twice a week, with an exercise professional, specifically trained in exercise oncology, supervising each session. Overall, the program followed the current recommendations for exercise in cancer provided by the American College of Sports Medicine (ACSM), further tailoring the prescription based on functional assessment and patient condition (12, 13). The exercise professional checked safety and adherence during the exercise program.

Over the 6 months, a combined aerobic and resistance training program was proposed, with the prescription organized into two macrocycles, each lasting 3 months (**Figure 1**). During the first three months, the primary focus was on increasing fat burn while maintaining or gaining muscle mass. In this sense, a moderated intensity continuous training was proposed. The duration of the aerobic part, performed on a treadmill or a cycloergometer, started with 15 minutes and progressively increased every two weeks until reaching 30 minutes per session. The intensity was set to a moderate level and checked using the rate of perceived exertion (RPE) - CR-10 Borg Scale, specifically at a 3–5 RPE. Resistance training included five bodyweight or elastic band exercises (e.g., squat, biceps curl, crunch, back extension, triceps extension, lunges), performed in 2–3 sets of 8–12 repetitions, which progressively increased over the weeks. Intensity was maintained at a moderate level, i.e., 3–5 RPE. A 5-minute warm-up (dynamic stretching) and cool-down (static stretching) preceded and concluded each session. Months 3–6 were dedicated to maintaining muscle mass and maximizing cardiorespiratory fitness. In this sense, the prescription of the aerobic component was reorganized into interval training, consisting of 3–4 rounds of 2–4 minutes at high intensity (5–7 RPE), interspersed with 1–3 minutes of light-intensity exercise (1–2 RPE). Progression in duration and number of rounds occurred over the week, based on the patient’s response. The resistance part was modified to incorporate progressive overload training, using isotonic machines or free weights, in 3–4 sets of 10–12 repetitions at a moderate to somewhat vigorous intensity, i.e., 3-6. The warm-up and cool-down remained equal.

Additionally, during the sessions, the patient was educated on maintaining an active lifestyle, especially on days off from training, and received suggestions to reduce and break up time spent on sedentary behaviors.

### 2.3 Assessments

Assessments were performed at baseline, after 3 and 6 months, by a researcher not involved in the delivery of the intervention. Before the physical fitness evaluations, blood pressure, heart rate, and oxygen saturation were recorded. The following physical fitness parameters were evaluated: i) functional capacity using the Six-Minute Walk Test, using the American Thoracic Society guidelines (14), ii) cardiorespiratory fitness, through the Astrand–Rhyming submaximal test (15), iii) muscle strength, using the handgrip strength test, iv) upper and lower limb muscular endurance, with the 30-second arm curl and the 30-Second Chair Stand Test (16, 17), v) anthropometric values, by body weight, height, and waist and hip circumferences (18), vi) body composition using the bioelectrical impedance scale (Tanita RD-545 HR), vii) static balance with the single leg stance test (19), viii) flexibility with the back scratch test and the chair sit-and-reach test (20). Patient-reported outcomes were collected using the following validated questionnaires: i) quality of life using the European Organisation for Research and Treatment of Cancer Quality of Life Questionnaire Core 30 (EORTC QLQ C-30) (21); ii) sarcopenia risk with the SARC-F (22); iii) fatigue level with the Brief Fatigue Inventory (23); iv) Godin’s Leisure Time Exercise Questionnaire to measure physical activity levels (24). Circulatory parameters were obtained from medical records. Additionally, routine staging CT images at the L3 vertebra level were used to quantify body composition changes since diagnosis and during the exercise program (December 2024, February 2025, May 2025).

## 3 Results

During the 6-month intervention, adherence to the sessions was 89.6%, with 5 exercise sessions missed due to personal reasons (vacation). Overall, the program was well-tolerated, with two mild adverse events (dizziness) recorded during the resistance training part.

**Table 1** displays the results of the physical fitness assessment. Body weight slightly decreased by 0.5 kg over the six months, as well as the BMI. Waist circumferences decreased by 6 centimeters (cm) during the first 3 months, and slightly increased by 1.5 cm at month 6, whereas hip circumferences reduced by 2 cm in the first 3 months and then remained stable. Bioimpedance analysis revealed an overall gain in muscle mass (+ 1.55 kg at 3 months (**Figure 2B**); +1.2 kg at 6 months) and a reduction in fat mass (-1.5 kg at 3 months; -1.8 kg at 6 months). The six-minute walking test remained stable over the 6 months, whereas estimated cardiorespiratory fitness increased (+3.9 ml/min/kg at 3 months; + 5.1 ml/min/kg at 6 months). Muscle strength increased in the first three months and then slightly decreased ([right arm; +8 kg at 3 months; +5 kg at 6 months]; [left arm; +3.5 kg at 3 months; -1 kg at 6 months]). Upper and lower limb muscular endurance improved at 3 and 6 months (right arm: +10 reps at 3 months; + 13 reps at 6 months; left arm: +9 reps at 3 months; + 11 reps at 6 months; chair sit-to-stand: +7 reps at 3 months; +10 at 6 months). Similarly, upper and lower limb flexibility was enhanced at 3 and 6 months (right arm: +0.5 cm at 3 months; +7.5 cm at 6 months; left arm: +4 cm at 3 months; +7 cm at 6 months; right leg: +15cm at 3 months; +24 cm at 6 months; left leg: +10 cm at 3 months; + 17 cm at 6 months).

**Table 1:** Absolute scores of physical fitness parameters.

Variables	At baseline	At 3 months	At 6 months
Blood pressure (mmHg)	130/88	140/85	123/76
Heart rate (bpm)	70	66	67
Saturation (%)	97	97	100
Anthropometric values			
Body weight (kg)	74.4	74.7	73.9
BMI (kg/m^2^)	24.2	24.2	23.9
Waist circumferences (cm)	88.0	82.5	84.0
Hip circumferences (cm)	102.0	100.0	100.5
Waist-hip ratio (cm)	0.86	0.82	0.83
Body composition			
Fat mass (kg)	19.0	17.5	17.2
Fat-free mass (kg)	55.5	57.15	56.6
Muscle mass (kg)	52.70	54.25	53.9
Functional capacity			
Six-minute walking test (m)	580.0	583.1	582.0
Cardiorespiratory fitness			
Astrand -Rhyming cycle ergometer (ml/min/kg)	26.0	29.9	31.1
Muscular strength			
Handgrip -leAT visceral adipose tissueft arm (kg)	43.0	46.5	42.0
Handgrip-right arm (kg)	42.5	50.5	47.5
Muscular endurance			
30s chair stand (repetitions)	13.0	20.0	23.0
30s arm curl-left arm (repetitions)	12.0	21.0	23.0
30s arm curl-right arm (repetitions)	14.0	24.0	27.0
Flexibility			
Back scratch-left arm (cm)	-14.0	-10.0	-7.0
Back scratch-right arm (cm)	-4.0	-3.5	+3.5
Chair sit and reach-left leg (cm)	-14.0	-4.0	+3.0
Chair sit and reach-right leg (cm)	-20.0	-5.0	+4.0

kg, kilograms; mmHg, millimeters of mercury; bpm, beats per minute; cm, centimeters; m, meters; ml/min/kg, milliliters per minute per kilogram.

The results of patient-reported outcomes are presented in **Table 2**. Among the patient-reported outcomes, improvements in several domains of quality of life were reported at 3 months: physical functioning (93.3 vs. 100 points), role functioning (50.0 vs. 100.0 points), emotional functioning 83.3 vs. 100.0 points), global health status 83.3 vs. 100.0 points) and pain (16.6 vs 0.0 points). At month 6, most domains remained stable, with a reduction in constipation observed compared to the baseline (33.3 vs. 0.0 points). The risk of sarcopenia remained stable and low throughout the study period, as well as the sleep habits and fatigue levels. The light-intensity physical activity decreased in the first three months and then increased (-15 minutes at 3 months; +70 minutes at 6 months); moderate-intensity activity increased in the first three months and then remained stable (+120 minutes at 3 and 6 months). No significant changes were observed for the circulatory parameters (**Supplementary Materials**). Notably, cholesterol and triglycerides remained above the normal range throughout the entire exercise intervention, with no significant modifications. Body composition, quantified through CT scans (**Figure 2A**), confirmed the bioimpedance results, reporting an increase in muscle area (160.4 vs. 164.6 cm^2^) and a decrease in visceral (89.9 vs. 84.4 cm^2^) and subcutaneous (151.3 vs. 133.0 cm^2^) adipose tissue.

**Table 2:** Absolute scores of patient-reported outcomes.

Variables	At baseline	At 3 months	At 6 months
Quality of life (score 0-100)			
Physical functioning	93.3	100.0	100.0
Role functioning	50.0	100.0	100.0
Emotional functioning	83.3	100.0	91.6
Cognitive functioning	100.0	100.0	100.0
Social functioning	100.0	100.0	100.0
Global health status	83.3	100.0	83.3
Fatigue	33.3	33.3	33.3
Nausea/vomiting	0	0	0
Pain	16.6	0	0
Dyspnea	0	33.3	0
Insomnia	0	0	0
Appetite loss	0	0	0
Constipation	33.3	33.3	0
Diarrhea	0	0	0
Financial problems	0	0	0
Sarcopenia risk			
* SARC-F (score 0-10)*	0	0	0
Sleep quality			
*Pittsburgh Sleep Quality Index* (0–21)	1.0	1.0	1.0
Subjective sleep quality (0–3)	0	0	0
Sleep latency (0–3)	0	0	0
Sleep duration (0-3)	0	0	0
Sleep efficiency (0-3)	0	0	0
Sleep disturbance (0-3)	1.0	1.0	1.0
Use of sleep medication (0-3)	0	0	0
Daytime dysfunction (0-3)	0	0	0
*Brief Fatigue Inventory (0-10)*	2.0	2.0	1.0
Physical activity level (minutes/week)			
Light	60.0	45.0	130.0
Moderate	0	120.0	120.0
Vigorous	0	0	0
Total	60.0	165.0	250.0

## 4 Discussion

To our knowledge, our case report represents the first evidence of a non-pharmacological approach in managing the well-known and common side effect of lorlatinib-induced weight gain (**Figure 2**). We found that a 6-month structured and supervised aerobic and resistance exercise intervention resulted in a slight decrease of 0.5 kilograms in overall body weight in a patient experiencing grade 1 weight gain. This could seem a partially positive result, given the relatively small loss of body weight. Nevertheless, the reported reduction in waist and hip circumferences may suggest a possible modulation in body composition. Effectively, the bioimpedance analysis showed a gain in muscle mass of +1.2 kg and a loss in body fat of -1.8 kg at the end of the training period. These results are further confirmed by the CT scan analysis, which reported an increase in muscle area and a reduction in both visceral and subcutaneous adipose tissue (**Figure 2**).

From this perspective, our results may have several important implications. On the one hand, the exercise intervention has demonstrated the ability to manage this side effect by stopping weight gain and, simultaneously, reducing fat mass (probably most impacted by lorlatinib). Indeed, an observational study on 46 ALK+ patients with NSCLC treated with ALK inhibitors has explored the body composition shaping caused by such oncological treatment. At 3 months, an increase of about 15% in visceral adipose tissue and of 12% in the subcutaneous adipose tissue was observed, while no modification in muscle mass was reported. These data further deteriorated 1 year after treatment started, where a gain of approximately 39% and 33% in visceral and subcutaneous adipose tissues, respectively, was noted (7). This investigation demonstrates not only that the ALK TKIs-induced weight gain is predominantly determined by an increase in fat mass, but also suggests the need for early intervention to manage this side effect, as it tends to worsen over time (7). Similarly, this case first described a rapid increase in fat mass shortly after the initiation of lorlatinib, as well as the efficacy of a prompt exercise-based intervention to manage this side effect, thereby counteracting the negative impact on body composition. This is particularly crucial, since it is easier to manage mild weight gain, even with a single supportive intervention, compared to a more severe one, where it is possible to speculate that exercise alone may have limited effectiveness. This may, indirectly, imply that intervention to manage ALK TKIs-induced weight gain should also be tailored according to the grade of the side effect, by proposing additional interventions, e.g., nutritional support, when the severity increases. On the other side, exercise improved the patient’s muscle mass. Skeletal muscle wasting is associated with poorer survival and worse treatment response in patients with NSCLC (25). Therefore, it is possible that exercise, beyond managing body composition, may also increase patient response to anticancer treatment. This observation is particularly relevant, considering that a recent study shows that higher at starting ALK-TKI drugs is correlated with higher toxicities leading to more dose reduction and treatment interruption (26); at the same time, preliminary findings on pharmacological options for managing weight loss, suggest a potential negative interaction with ALK inhibitors (24), further supporting the adoption of a non-pharmacological approach, such as exercise, as a first-line intervention to manage lorlatinib-induced weight gain.

Additionally, we found that exercise improved patients’ physical fitness, including cardiorespiratory fitness, muscle strength, and endurance, as well as enhanced various reported outcomes, such as quality of life and the amount of physical activity. These findings align with the current literature, which emphasizes the beneficial effects of exercise on these outcomes in patients with lung cancer (10). However, it also provides novel information, due to the currently limited evidence regarding the impact of exercise in the context of TKI treatment. Optimizing cardiorespiratory fitness and muscle strength in patients with cancer is gaining increasing importance. A systematic review with meta-analysis, including 42 studies and more than 46,000 patients with cancer, has shown that high levels of cardiorespiratory fitness and muscle strength are associated with a reduction of 44% and 31% respectively, in the risk of all-cause mortality (27). At the same time, preserving the quality of life is a crucial aspect and can be considered a primary goal when a curative intent is not possible, such as in the context of metastatic disease.

Among the limitations, the absence of follow-up data limits our ability to assess whether the observed benefits were maintained over time. Additionally, the design of the case report limits the generalizability of the findings, despite the patient reflecting the clinical and biological characteristics of patients affected by oncogene addicted NSCLC.

Several questions about weight gain and TKI remain open, such as exploring the determinants and consequences of such gain. In this sense, we have designed an observational study (Protocol n: 647CET; BOLT study) aimed at quantifying the impact of different TKIs on body composition, as well as exploring the impact of this side effect on clinical outcomes. Moreover, the collection of data regarding physical activity, nutritional intake, and blood parameters will permit the analysis of other determinants potentially contributing to the onset of weight gain, consequently proposing more tailored interventions to manage it.

In conclusion, we report that a supervised 6-month exercise intervention was a safe and effective option for managing grade 1 lorlatinib-induced weight gain in a patient with ALK+ NSCLC. This case may represent the backbone for further interventional studies aimed at determining the real efficacy of such intervention in preventing or controlling weight gain in this population.

## Data Availability

The original contributions presented in the study are included in the article/**Supplementary Material**. Further inquiries can be directed to the corresponding author.
