# Supplementary material for: Managing lorlatinib-induced weight gain through a structured exercise intervention in an ALK+ NSCLC patient: a case report
Source: Front Oncol. 2025 Nov 20;15:1672319. doi: 10.3389/fonc.2025.1672319 (PMC12675186; doi:10.3389/fonc.2025.1672319)
Supplement: Supplementary file 1 [file Table1.docx]

**Table S1.** Blood parameters at baseline, after 3 and 6 months of the exercise program

| Variable | At baseline | After 3 months | After 6 months | Normality range |
| --- | --- | --- | --- | --- |
| Erythrocytes (10^12^/L) | 5.39 | 4.98 | 5.05 | [4.00 - 5.20] |
| Hemoglobin (g/L) | 146 | 148 | 145 | [135 - 175] |
| MCV (fL) | 85.3 | 90.8 | 88.1 | [80.0 - 99.0] |
| MCH (pg) | 27.1 | 29.7 | 28.7 | [26 - 34] |
| MCHC (g/L) | 317 | 327 | 326 | [310 - 360] |
| RDW (%) | 14 | 12.2 | 11.9 | [11.5 - 15.0] |
| Platelets (10^9^/L) | 140 | 133 | 119 | [150 - 400] |
| Erythroblasts (10^9^/L) | Absent | Absent | Absent | Absent |
| MPV (fL) | 13 | 13 | 13 | [9.6 - 12.9] |
| Leukocytes (10^9^/L) | 6.47 | 5.89 | 5.98 | [4.30 - 10.0] |
| Neutrophils (10^9^/L) | 2.33 | 1.87 | 2.22 | [1.80 - 8.00] |
| Lymphocytes (10^9^/L) | 3.15 | 3.17 | 2.80 | [1.20 - 4.00] |
| Monocytes (10^9^/L) | 0.59 | 0.54 | 0.44 | [0.20 - 1.00] |
| Eosinophils (10^9^/L) | 0.37 | 0.28 | 0.50 | [< 0.45] |
| Basophils (10^9^/L) | 0.03 | 0.03 | 0.02 | [< 0.20] |
| Urea (mg/dL) | 34 | - | 32 | [17.1 - 47.1] |
| Creatinine (µmol/L) | 83 | 89 | 85 | [44.0 - 106.0] |
| Bilirubin (µmol/L) | 4.8 | 4.1 | 5.3 | Less than 18.0 |
| Calcium (mmol/L) | 2.38 | 2.49 | 2.42 | [2.10 - 2.60] |
| Sodium (mmol/L) | 142 | 142 | 139 | [135 - 145] |
| Potassium (mmol/L) | 3.81 | 3.93 | 3.68 | [3.40 - 4.80] |
| Glucose (mg/dL) | 86 | 82 | 91 | [60 - 99] |
| P-ast (U/L) | 42 | 40 | 37 | [5 - 45] |
| P-alt (U/L) | 64 | 55 | 48 | [6 - 45] |
| P-alp (U/L) | 63 | - | 66 | [50 - 130] |
| Cholesterol (mg/dL) | 215 | 207 | 232 | Less than 200 |
| Triglycerides (mg/dL) | 249 | 226 | 255 | Less than 150 |
